# A systematic review of the clinical evidence for an association between type I hypersensitivity and inner ear disorders

**DOI:** 10.3389/fneur.2024.1378276

**Published:** 2024-03-25

**Authors:** Bin Zeng, Ewa Domarecka, Lingyi Kong, Heidi Olze, Jörg Scheffel, Sherezade Moñino-Romero, Frank Siebenhaar, Agnieszka J. Szczepek

**Affiliations:** ^1^Department of Otorhinolaryngology, Head and Neck Surgery, Charité - Universitätsmedizin Berlin, Corporate Member of Freie Universität Berlin and Humboldt Universität zu Berlin, Berlin, Germany; ^2^Institute of Allergology, Charité - Universitätsmedizin Berlin, Corporate Member of Freie Universität Berlin and Humboldt-Universität zu Berlin, Berlin, Germany; ^3^Fraunhofer Institute for Translational Medicine and Pharmacology ITMP, Allergology and Immunology, Berlin, Germany; ^4^Institute of Health Sciences, Collegium Medicum, University of Zielona Góra, Zielona Góra, Poland

**Keywords:** IgE, allergy, hearing loss, vertigo, endolymphatic hydrops, Ménière’s disease, sudden sensorineural hearing loss, acute low-tone sensorineural hearing loss

## Abstract

Inner ear disorders have a variety of causes, and many factors can contribute to the exacerbation of cochlear and vestibular pathology. This systematic review aimed to analyze clinical data on the coexistence and potential causal interaction between allergic diseases and inner ear conditions. A search of PubMed and Web of Science identified 724 articles, of which 21 were selected for full-text analysis based on inclusion and exclusion criteria. The epidemiologic evidence found overwhelmingly supports an association between allergic disease and particular inner ear disorders represented by a high prevalence of allergic reactions in some patients with Ménière’s disease (MD), idiopathic sudden sensorineural hearing loss (ISSHL), and acute low-tone hearing loss (ALHL). In addition, patients with MD, ISSHL, and ALHL had higher levels of total serum IgE than healthy subjects. Finally, in some cases, changes in cochlear potential may have been induced by antigen exposure, while desensitization alleviated allergy and inner ear-related symptoms. The exact mechanism of interaction between the auditory/vestibular and immune systems is not fully understood, and further clinical and basic research is needed to understand the relationship between the two systems fully.

## 1 Introduction

The mammalian inner ear is a structure that allows auditory and balance perceptions. Located in the temporal bone, it consists of the cochlea, responsible for the auditory function, semi-circular canals that sense a rotary motion, and the vestibule comprising utricle and saccule that detect forward or backward and downward or upward movements, respectively (1). Another component of the inner ear is the endolymphatic sac, which is an extension of the cochlear duct and is responsible for adsorbing the endolymph produced by the cochlear stria vascularis and the dark cells of the vestibule (1). Multiple disorders can affect the inner ear, resulting in hearing loss and tinnitus (2) or dizziness (3). Some of these conditions are associated with exposure to ototoxic substances [e.g., heavy metals or organic solvents (4)] or ototoxic medications [e.g., aminoglycoside antibiotics or cisplatin (5)], some others with noise exposure (noise-induced hearing loss (6)) and aging [presbyacusis (7)]. Interestingly, hearing loss can also be caused by an autoimmune inner ear disease (AIED), a rare condition associated with bilateral or asymmetric progressive hearing loss and the presence of autoantibodies or autoreactive T cells in the circulation (8, 9). Finally, hereditary hearing and balance disorders can be non-syndromic or syndromic, such as Usher syndrome (10) or Alport syndrome (11).

Among the less understood inner ear disorders are Ménière’s disease (MD), idiopathic sudden sensorineural hearing loss (ISSHL), and acute low tone hearing loss (ALHL). All three of these disorders affect patients’ ability to communicate and, over time, make them candidates for cochlear implantation. MD is a disorder that affects the entire inner ear, negatively impacting hearing and balance and manifesting as episodes of vertigo and fluctuating hearing symptoms: low- to mid-frequency sensorineural hearing loss, tinnitus, and fullness in the ear (12). Having an estimated prevalence of 0.19% among insured persons in the USA, MD is reported mainly by adults (commonly aged 40–60 years), and only about 3% of cases occur in persons younger than 18 (13–16). As the number of episodes increases, the fluctuating hearing symptoms get more pronounced and permanent, resulting in unilateral deafness (17). Unfortunately, about half of the patients with unilateral MD develop contralateral disease over time, ultimately leaving them with bilateral deafness (17). Research into understanding the pathological mechanism leading to MD began almost a century ago. In 1938, the histopathological findings in temporal bones of patients with MD were described independently by Hallpike in England (18) and Yamakawa in Japan (19). Both groups found endolymphatic hydrops, which refer to a swelling of the cochlear duct (scala media) in the MD patients (20). Endolymphatic hydrops may lead to the temporary rupture of the Reissner membrane, resulting in the mixing of endolymph and perilymph and a decrease in the endocochlear potential, leading to clinical symptoms. Although the endolymphatic hydrops in patients with MD is characteristic of this disease, its etiology has not yet been well elucidated. Contemporary treatment of MD is mainly symptomatic and involves dietary restrictions, intratympanic injection of steroid or ototoxic medications, systemic use of betahistine, diuretics, vestibular neurectomy, or endolymphatic sac surgery (17). The concept of allergy being one of the factors contributing to MD’s pathogenesis was first proposed by Duke (21). Over the past decades, researchers have studied and reviewed this interesting hypothesis (22–27).

ISSHL is a unilateral auditory condition accompanied by fullness in the ear and tinnitus and is sometimes associated with vestibular symptoms. ISSHL has an incidence of 5–20 per 100,000 people annually (28). The pathophysiology of this disease remains largely unknown. In some cases, the reasons might be tracked to vestibular schwannoma, multiple sclerosis, stroke, infections of the central nervous system, and others. Even though spontaneous recovery may occur, local or systemic corticosteroid therapy is often used as the treatment of choice. However, only a proportion of treated patients recover (28). The hypothesis about the role of allergy in the etiopathogenesis of ISSHL was posed in 2006 by Lombardi et al. (29), who suggested that local (cochlear) allergic inflammation could cause SSHL. ALHL is often viewed as a subtype of ISSHL, with the main difference being that it does not affect high but low frequencies. Some ALHL patients develop tinnitus and vertigo and progress to MD over the years (30). The transformation of ALHL to MD has recently been associated with elevated serum IgE antibody levels, suggesting a possible association with allergy (31). In addition, the frequent positive response of ALHL patients to corticosterone therapy was suggested to link this type of hearing loss and the immune system (32, 33).

Allergic reactions refer to undesirable responses of the immune system and are classified into four types of hypersensitivity (34): Type I: immediate hypersensitivity or anaphylaxis; Type II: cytotoxic or antibody-dependent hypersensitivity; Type III: immune complex disease and Type IV: delayed-type hypersensitivity. Type I hypersensitivity is a rapid allergic reaction provoked by re-exposure to a specific type of antigen, with hay fever or allergic asthma as classic examples. It is driven by the IgE class of antibodies specific to a particular antigen bound to mast cells or basophils via the high affinity receptor for IgE, FcεRI. Cross-linking of the receptor by an antigen induces their degranulation and rapid release of inflammatory mediators including histamine and various proteases. Common environmental and food allergens are typical triggers of type I hypersensitivity reactions.

In recent years, there have been reports of immune system cells residing in the inner ear of humans and animals under steady-state conditions. These include lymphocytes, macrophages (35, 36), neutrophils, B-, T-, NK-, myeloid cells (33), and mast cells (37, 38). Immune cells can also infiltrate the cochlea from the periphery after noise exposure (39). The precise role of resident immune cells is not well understood. Still, the ability of peripheral myeloid-derived cells to colonize or infiltrate the inner ear suggests the existence of an interconnection between the auditory sensory and immune systems.

In the present study, we asked if there a relationship between allergic diseases and inner ear function. To answer this question, we conducted an extensive literature search to identify evidence of a possible association between allergies and inner ear disorders, or lack thereof. We were interested in which inner ear disorders were observed to be comorbid with allergic disorders and which allergic disorders were observed to be comorbid with inner ear disorders. We were also interested in how the hearing or vestibular system responds to treating allergic diseases in comorbid patients.

## 2 Materials and methods

### 2.1 Search strategy

This study’s literature review was conducted in June 2023, using two databanks: the US National Library of Medicine – National Institutes of Health (PubMed) and the Web of Science. The keywords included the following keywords:

- “Mast cell” AND “hearing loss” OR “Vertigo” OR “Tinnitus” OR “Endolymphatic hydrops” OR “Ménière disease”
- “IgE” AND “hearing loss” OR “Vertigo” OR “Tinnitus” OR “Endolymphatic hydrops” OR “Ménière disease”
- “Allergy” AND “hearing loss” OR “Vertigo” OR “Tinnitus” OR “Endolymphatic hydrops” OR “Ménière disease”

Full-text articles were downloaded when the title, abstract, or keywords indicated that the study qualifies for this study.

### 2.2 Selection criteria

#### 2.2.1 Inclusion criteria

- Articles published before June 2023
- Original research

#### 2.2.2 Exclusion criteria

- Literature review, case report, letter to Editor, and editorials
- Full text not available (no online access, or the local library was unable to obtain a copy via library networks)
- Articles written in a language other than English
- Animal studies

**Figure 1:**
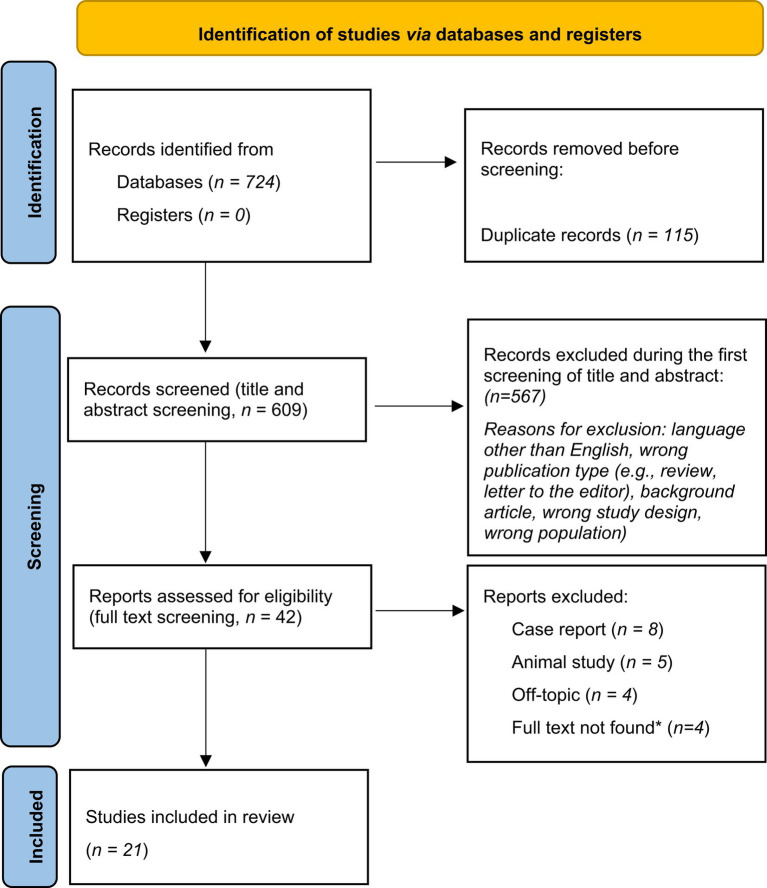
The PRISMA diagram (40) visualizing the literature selection process. “n” signifies the number of publications. *(Full text not found): the publication was not available online, and the local library and national library network could not provide a reprint.

Our search identified 724 studies in total. After removing duplicate studies, two individual reviewers independently screened the titles and abstracts of the remaining 609 articles. After applying the selection criteria, 21 articles were included in the analyses (Figure 1).

## 3 Results

### 3.1 Characteristics of included studies

The research teams that published the 21 articles included in this review were based in Europe, North America, Asia, and Africa. Seventeen publications (81%) had open or free access.

Twenty studies used a prospective design, one used a retrospective design, and 15 used case controls (Table 1). Fifteen studies were purely observational, whereas six applied anti-allergic interventions or antigen challenges to observe otologic changes; 16 focused on the characterization and presence of allergies in patients with otologic conditions, and five addressed the presence of otologic conditions in allergic patients. Only four papers were published in the last 5 years, while the rest were published between 1992 and 2013.

**Table 1:** Characteristics of the studies included in this review.

References	Design	Case-controlled	Type of study	Study focus	Disease studied	How was the information about inner ear Status obtained	How was the information about immune status obtained
Viscomi and Bojrab (41)	Prospective	No	Immune challenge/interventional	Allergies in otologic patients	MD	Pure-tone audiometry, speech discrimination, ECochG	Medical history, challenge with allergen
Gibbs et al. (42)	Prospective	No	Immune challenge/interventional	Allergies in otologic patients	MD	ECochG	Medical history, challenge with allergen
Derebery (43)	Prospective	Yes	Immune challenge/interventional	Allergies in otologic patients	MD	Not stated	Questionnaire
Noell et al. (44)	Prospective	Yes	Immune challenge/interventional	Allergies in otologic patients	MD	ECochG	Medical history, RAST
Eaton et al. (45)	Prospective	No	Immune challenge/interventional	Allergies in otologic patients	MD	ECochG	Medical history, RAST
Topuz et al. (46)	Prospective	No	Immune challenge/interventional	Allergies in otologic patients	MD	Pure-tone audiometry, speech discrimination, ECochG, reporting inner ear symptoms	SPT, challenge with allergen
Howard et al. (47)	Prospective	No	Observational	Allergies in otologic patients	MD	Medical history	Medical history, RAST, total IgE levels
Derebery and Berliner (48)	Prospective	Yes	Observational	Allergies in otologic patients	MD	Medical history	Survey
Boulassel et al. (49)	Prospective	Yes	Observational	Allergies in otologic patients	MD	Medical history	ELISA (antigen—food allergens, detection—atni-IgG, anti-IgA)
Keles et al. (50)	Prospective	Yes	Observational	Allergies in otologic patients	MD	Medical history	FACS analysis of PBMCs, cytokine ELISA
Toubi et al. (51)	Prospective	Yes	Observational	Allergies in otologic patients	ISSHL	Medical history	Indirect IFA, SPT, total IgE
Sen et al. (52)	Prospective	Yes	Observational	Allergies in otologic patients	MD	Questionnaire	Questionnaire
Di Berardino and Cesarani (53)	Prospective	Yes	Observational	Allergies in otologic patients	MD	Medical history	SPT
Keles et al. (54)	Prospective	Yes	Observational	Allergies in otologic patients	ISSHL	Medical history	Flow cytometry, ELISA (total IgE, IL4, IL10, and IFN-γ), SPT
Ma et al. (31)	Prospective	Yes	Observational	Allergies in otologic patients	ALHL	Medical history, ECochG	Total IgE, specific IgE
Roomiani et al. (55)	Prospective	Yes	Observational	Allergies in otologic patients	MD	Medical history	Total IgE, specific IgE
Lasisi and Abdullahi (15)	Retrospective	No	Observational	Otologic conditions in allergy patients	AR	Medical records	Medical records
Singh et al. (56)	Prospective	Yes	Observational	Otologic conditions in allergy patients	AR	Pure-tone audiometry with extended high frequencies (0.250–16 kHz), tympanometry, OAE, ABR	Medical records
Karabulut et al. (57)	Prospective	Yes	Observational	Otologic conditions in allergy patients	AR	Pure-tone audiometry	SPT, medical records
Sahni et al. (58)	Prospective	Yes	Observational	Otologic conditions in allergy patients	AR	Pure-tone audiometry, OAE	Medical records
Mahajan et al. (59)	Prospective	Yes	Observational	Otologic conditions in allergy patients	AR	Pure-tone audiometry OAE, ABR	Medical records

Studies were sorted by publication date. MD, Ménière’s disease; ISSHL, idiopathic sudden sensorineural hearing loss; ALHL, acute low tone sensorineural hearing loss; AR, allergic rhinitis; IFA, immuno-fluorescence assay; RAST, radioallergosorbent test; SPT, skin prick testing; ECochG, electrocochleography; OAE, otoacoustic emissions; ABR, auditory brainstem responses.

Ten publications examined the epidemiologic relationship between allergy and inner ear disease, six studied the immunopathologic relationship, one article focused on the effect of allergy treatment on MD, and four articles used electrocochleography to document changes in inner ear function after intranasal challenge in patients with MD.

The sex ratio and age range of study participants varied among the included studies. Thirteen (62%) articles had a female/male ratio > 1 (range 1.1:1–3.8:1), one (5%) article had a female/male ratio 1:1, four (19%) had a female/male ratio < 1 (range 0.3:1–0.9:1) and three articles did not report gender. Regarding the age range, 17 (80%) of the studies enrolled subjects between 30 and 60 years, two (10%) included subjects younger than 30 years, and the other two did not mention the age of the patients. Medians and interquartile ranges (IQRs) could not be calculated because age reporting varied between articles, ranging from reporting only the mean and standard deviation to reporting the age of individual patients to reporting only the age range.

### 3.2 Epidemiology

Ten studies focused on the epidemiological aspect of the association between allergy and inner ear disease (Table 2). Four of these studies examined hearing function in patients diagnosed with allergic rhinitis (AR)/nasal allergy, while the other six focused on the prevalence of allergy in patients with inner ear conditions.

**Table 2:** Characteristics of studies linking allergy and inner ear disease. The number of patients and controls is shown, and the main findings are presented.

References	Study group	Control group	Main findings
Howard et al. (47)	Patients with otologic disorders, *n* = 186	None	38% of patients had evidence of inhalant allergy
Derebery and and Berliner (48)	MD patients, *n* = 734	Patients with otologic problems than MD, *n* = 172	59.2% of MD patients reported possible airborne allergy, 40.3% had or suspected food allergies, and 37% had had confirmatory skin or *in vitro* tests for allergy. These prevalence rates were significantly higher than those found in the control group, of which 42.7% reported having or suspecting airborne allergies, and 25% had or suspected food allergies (*p* ≤ 0.005)
Keles et al. (50)	MD patients, *n* = 48	Healthy volunteers, *n* = 46	Allergy history was found in 67.3% of the patients, while the ratio in the control group was 34.7%
Sen et al. (52)	MD, *n* = 108	Other otologic problems, *n* = 100	52% of patients with MD reported allergy, which was significantly higher than allergy in the control group (23%, *p* < 0.001)
Topuz et al. (46)	MD, *n* = 48	None	After the provocation, 30 (62.5%) patients had tinnitus and fullness in the diseased ear, and 6 (12.5%) patients had vertigo
Lasisi and Abdullahi (15)	AR, *n* = 144	None	Otologic symptoms found in 66% of subjects with AR: peripheral vestibular symptoms11.6%; idiopathic tinnitus 26.3%; idiopathic SHL 18%; cochlear hydrops 6%; autoimmune inner ear disease 6%
Singh et al. (56)	AR, *n* = 30	Healthy volunteers, *n* = 20	There is a higher prevalence of hearing loss and otoacoustic emission abnormalities in the AR group than in the controls
Di Berardino and Cesarani (53)	MD, *n* = 58	Healthy volunteers, *n* = 25 Multiple allergens sensitized patients, *n* = 25	82.7% of MD patients were positive for skin prick tests for one or more allergens
Sahni et al. (58)	AR, *n* = 100	Healthy volunteers, *n* = 100	32% of AR patients had abnormal OAE
Mahajan et al. (59)	AR, *n* = 100	Healthy volunteers, *n* = 50	AR patients had abnormal OAE in the frequencies between 1,481 and 8,000 Hz and identified prolonged wave I in ABR

MD, Ménière’s disease; BPPV, Benign paroxysmal positional vertigo; SHL, sensorineural hearing loss; AR, allergic rhinitis; ABR, auditory brainstem response; DPOAE, distortion product otoacoustic emission; OAE, otoacoustic emission.

A study published in 1997 by Howard et al. (47) analyzed the data of 186 patients seen for dizziness (66%), tinnitus (63%), hearing loss (49%), ear fullness (48%), MD (27%), balance disorders other than dizziness (21%), and Eustachian tube dysfunction (4%). The authors found a 38% prevalence of immunoglobulin E-mediated hypersensitivity in a group of patients seen for neuro-otologic symptoms, not sinus symptoms. This prevalence was within the range for unspecified rhinitis (14–63.3%) or AR (3.5–54.5%) reported in the United States (60). Later, in 2000, Derebery et al. found the prevalence of airborne allergy among MD patients to be 41.6% (*n* = 734), which was higher than in the general population (31.5%) or in the population of patients attending an otologic clinic for other symptoms (27.6%; *n* = 172) (48). This observation was supported in 2005 by Sen et al. (52), who showed by questionnaire that the prevalence of allergy in patients with MD was 51.9%, which was significantly higher than in the age- and sex-matched controls (23%; *p* < 0.05) or that found in the general population. Approximately half of the allergies reported by patients with MD were attributed to food allergies, and the other half to airborne allergies. Topuz et al. found that all 48 MD patients included in the study were atopic according to skin prick testing (SPT) for food and inhalant allergens (46). Importantly, none of the patients tested had noticed allergic symptoms. Finally, Di Berardino et al. performed SPT in patients with definite MD. The antigens used included several common inhalants, bovine milk proteins, ovalbumin, wheat flour, tomato, potato, apple, carrot, and gliadin (53). This study showed 82.7% of MD patients were positive for one or more allergens.

Four studies have investigated the prevalence of hearing dysfunction in patients with allergies. Lasisi et al. (15) performed a retrospective analysis of patients with known nasal allergies. In this study, clinical diagnoses of inner ear disorders were made in 95 of 144 patients included in the study (66%) and comprised 25 cases of idiopathic tinnitus (17.4%), 17 cases of idiopathic sensorineural hearing loss (SHL) (11.8%), 13 cases of idiopathic vertigo (9%), 11 cases of benign paroxysmal positional vertigo (7.8%), 9 cases of MD (6.3%), 8 cases of vestibular hydrops (5.6%), 6 cases of cochlear hydrops (4.2%), and 6 cases of autoimmune inner ear disease (4.2%). A recent umbrella review estimated the pooled prevalence of diagnosed tinnitus in adults worldwide to be 3.4% (61), so the diagnosis of idiopathic tinnitus in 17.4% of patients with allergies indicates a higher prevalence than in the general population. It is complex to compare the prevalence of SHL shown in the study by Lasisi et al. with the general population because the authors did not specify which subtype of sensorineural hearing loss was diagnosed (e.g., sudden, age-related, or noise-induced). The prevalence of idiopathic vertigo in the general population has been estimated to be 7% (62), close to the 9% observed in the study. However, the prevalence of benign paroxysmal positional vertigo in allergic patients (7.8%) was higher than the lifetime prevalence (2.4%) or the 1-year prevalence (1.6%) in the general population (63). The prevalence of MD in the general population ranges from 0.0035 to 0.513% (17). In contrast, the general population’s epidemiology of vestibular and cochlear hydrops remains unknown, but they are often associated with MD. Therefore, the reported prevalence of MD (6.3%) and both types of hydrops (5.6 and 4.2%) is higher than expected in the general population. Finally, autoimmune inner ear disease is also rare (incidence of 0.015%) (64), and its detection in 4.2% of allergy sufferers suggests that it is more common in this group of patients. In summary, the prevalence of inner ear disease was higher in Lasisi’s study than in the general population.

Singh et al. (56) used inner ear-specific tests to compare the hearing abilities of 30 patients with AR and 20 age- and sex-matched healthy controls. Tests included otoacoustic emissions (OAE) which measure a byproduct of sound processing by the cochlea, and auditory brainstem response (ABR) which records the electrical activity occurring in eight cranial nerve and the auditory brainstem in response to sound stimulation. They found that 90% of AR patients had abnormal OAE in the frequencies between 1,003 and 3,991 Hz, consistent with cochlear damage. This was in contrast to healthy (AR-free) controls. In addition, there were also significant differences in the ABR, such as prolongation of the wave I latency, indicating cochlear damage in the AR patients, which was absent in the controls who had wave I values within the norm (65). von Brevern et al. (63) used the same audiometric tests to evaluate the hearing ability of 100 AR patients and 50 controls. They also found that AR patients had abnormal OAE in the frequencies between 1,481 and 8,000 Hz and identified prolonged wave I in ABR. Finally, in a sample of 100 AR patients and 100 controls, Sahni et al. (58) found that 35% had abnormal OAE compared to only 7% of controls. Despite the differences in the sample composition (age ranges: Singh et al. 17–45; Sahni et a. 10–40; Mahajani’s study provided only the mean age, which was 32.91 SD =12.40), which could have contributed to the differences seen, the primary trend of the findings supports the evidence that AR patients have a higher prevalence of ABR and OAE abnormalities consistent with sensorineural hearing loss than healthy controls.

### 3.3 Immunopathology

Six studies (28.6%) included in this review investigated immune parameters in patients with inner ear disease (Table 3). Boulassel et al. (49), Keles et al. (50), and Roomiani et al. (55) focused on MD patients. Keles et al. and Roomiani et al. showed that MD patients had a higher concentration of total serum IgE than healthy subjects or patients with otologic disorders other than MD. Keles also investigated cytokine profiles, allergic parameters, and lymphocyte subsets in blood and serum of 46 MD patients and 46 age-matched healthy controls. This study found that the mean level of total IgE in serum was significantly higher in MD patients (mean IgE concentration 267.10 ± 394.69 IU/mL) than in the controls (mean IgE concentration 97.78 ± 87.34, *p* < 0.05) and that there were more subjects in MD group with elevated total IgE (19/46, 41.3%) than in the control group (9/46, 19.5%). Furthermore, MD patients had more circulating CD4^+^ cells (mean 44.22% ± 8.86) than controls (mean 39.49 ± 4.85; *p* < 0.05), increased CD4^+^/CD8^+^ cell ratio (MD 1.65 ± 0.71, controls 1.22 ± 0.24; *p* < 0.05), more circulating CD23^+^ cells (MD mean 10.09% ± 3.96, controls 6.82% ± 1.88; *p* < 0.05), and elevated blood concentrations of interleukin–4 (IL-4) (MD mean IL4 6.35 pg./mL ± 6.20, controls 2.67 pg./mL ± 0.65; *p* < 0.05). The test results for specific IgE levels were grouped into four categories: negative, mildly positive (specific IgE levels between 3.5 and 17.5 IU/mL), positive (specific IgE levels between 17.5–50.0 IU/mL) and strongly positive (greater than 50 IU/mL). Interestingly, when tested for specific IgE levels, several MD patients were strongly positive for fungi (6, 13.0% of the group), fruit (2, 4. 3% of the group), cow’s milk (12, 26.0% of the group), wheat flour (2, 4.3% of the group), beef (2, 4.3% of the group), and rice (4, 8.6% of the group), while none of the controls tested strongly positive for any of the allergens. In addition, positive correlations were found between the counts of CD23^+^ cells and IgE, CD8^+^ cells and IgE, CD4^+^/CD8^+^ ratio and IgE, and CD23^+^ and CD8^+^ cells (*p* < 0.01 for each correlation).

**Table 3:** Studies on the immunopathologic link between allergy and inner ear disorders.

References	Study group	Control group	Main findings
Boulassel et al. (49)	MD, *n* = 29	Healthy volunteers, *n* = 29	Lack of significant increase in IgG or IgA antibodies against any common food allergen
Keles et al. (50)	MD, *n* = 48	Healthy volunteers, *n* = 46	In MD patients: positive correlations between: CD23 and IgE, CD8 and IgE, CD4/CD8 and IgE, and CD23 and CD8. Elevated total IgE. Increased numbers of CD4^+^ and CD23^+^ cells, greater CD4/CD8 ratio, increased IL-4
Toubi et al. (51)	ISSHL, *n* = 51	Healthy volunteers, *n* = 35	Significantly more ISSHL patients than controls have antinuclear and anti-thyroid antibodies. Presence of rheumatoid factor in 12%; anti-CL antibodies in 31%, anti-Bz GPI antibodies in 12% of ISSHL. Elevated total IgE in 27% and positive skin tests in 42% of patients.
Keles et al. (54)	ISSHL, *n* = 31	Healthy volunteers, *n* = 30	ISSHL group had positive correlations between IgE and CD23, IL10, IL4; IL4 and IL10, CD23^+^ and CD4^+^ cells; IL10 and CD23, CD4 (*p* = 0.000); 25.8% of patients had elevated total IgE levels, and 61.9% had a history of allergy
Ma et al. (31)	ALHL, *n* = 115	ISSHL, *n* = 127	Total IgE and specific IgE levels correlated with the SP/AP ratio in the ECochG of the ALHL group
Roomiani et al. (55)	MD, *n* = 39	Healthy volunteers, *n* = 41	Elevated total IgE serum level in MD patients. Significant correlation between MD symptoms and serum immunoreactivity to allergens

MD, Ménière’s disease; SHL, sensorineural hearing loss; ISSHL, idiopathic sudden sensorineural hearing loss; ALHL, acute low tone sensorineural hearing loss; ECochG, electrocochleography.

Boulassel et al. (49), however, showed that MD patients had no significant increase in specific IgG and IgA antibodies against common allergens [gliadin, ß-lactoglobulin, soy, albumin, ovalbumin, DPT (house dust mite), and *S. cerevisiae*]. Yet, in contrast to the other publications, Boulassel et al. determined antibody titers in serum by ELISA, and did not used SPT to identify IgE sensitization.

Three other studies focused on the association between idiopathic sudden sensorineural hearing loss (SSHL or ISSHL) and allergies. Consistent with the hypothesis of Lombardi et al. (29), Keles et al. evaluated the role of allergy in SSHL by serum cytokine profile, allergic parameters, and characterization of lymphocyte subsets in a sample of 31 SSHL patients and 30 age-matched healthy control subjects (54). The SSHL patients had a significantly higher percentage of CD23^+^ circulating lymphocytes (median 9.05, range 4.82–17.15) than the controls (median 5.40, range 2.50–18.17), *p* < 0.001, as well as CD4^+^ cells (SSHL patients median 43.7%, range 36.32–58.37; controls median 38.60, range 30.10–49.10; *p* < 0.001). In addition, in SSHL patients, strong positive correlations were found between the IgE levels, the numbers of CD23^+^ cells, and the serum concentrations of IL-4 and IL-10 (*p* < 0.001). IL-4 and IL-10 concentrations correlated and with numbers of CD23^+^ and CD4^+^ cells *p* = 0.000. This finding was consistent with a prospective follow-up study by Toubi et al. (51). In this study, 27% of SSHL patients (14/51) had significantly higher levels of total IgE compared to 8% of controls (3/35); *p* = 0.03. Six of the 14 patients with elevated IgE had a positive SPT for at least one allergen, but only three reported clinical symptoms. In the study of Ma et al., which included 115 patients with acute low-tone sensorineural hearing loss (ALHL) and 127 subjects with “conventional,” high-frequency idiopathic SSHL, differences in total and specific IgE levels were found between these two groups (31). ALHL patients had a mean of 66.47 IU/mL of total IgE (IQR 24.56–180.96), whereas SSHL patients had 27.01 IU/mL of total IgE (IQR 6.76–84.25), *p* = 0.000. Moreover, the levels of specific IgE significantly differed between the groups, being 9.42 AU/mL in the ALHL group (IQR 1.42–22.23) and 0.68 AU/mL in the SSHL group (IQR 0.39–2.6), *p* = 0.000.

The electrocochleography (ECochG), provides an objective information about the summation potential (SP, reflecting distortion products from outer hair cells) and action potential (AP, reflecting fluid balance in the inner ear) in the cochlea. An elevated SP/AP ratio indicates endolymphatic hydrops (41). In the ALHL patients, the IgE levels positively correlated with the SP/AP ratio measured by ECochG (multiple regression model *R*^2^ = 0.413, *p* = 0.001 for specific IgE, *p* = 0.001 for total IgE) (31).

### 3.4 Impact of antigenic stimulation or allergy treatment on inner ear disorders

We identified seven studies in which allergic challenges and /or anti-allergy interventions were used, and the outcome measures were otologic tests (Table 1). In the first study in 1992, Viscomi and Bojrab (41) monitored inner ear responses to antigenic stimulation in five MD patients using ECochG. All of the patients had a high SP/AP when they were challenged by intracutaneous injection with the antigens to which they were allergic, which included milk, wheat, soy, egg, and corn. In a study conducted by Howard et al. (47), patients with allergies were identified by a radioallergosorbent test (RAST) testing of 186 individuals with otologic disorders that included tinnitus, vertigo, fluctuating hearing loss, aural fullness, MD, perilymphatic fistula, multiple sclerosis, and, and posterior cranial fossa circulation compromise. Of the 186 subjects, 78 (36%) were RAST-positive. From seven MD patients with proven IgE-mediated allergy who agreed to immunotherapy (protocol not described in the paper), all reported a subjective improvement in their otologic symptoms. In another study performed in 1999 by Gibbs et al. (42), seven MD patients with known inhalant allergies were identified and challenged via the nasal mucosa for 20 min with patient-specific allergens (not specified). ECochG performed after the challenge indicated an SP/AP ratio elevation in four of seven MD patients. In 2001, Noell et al. (44) followed the study of Gibbs et al. and identified eleven MD patients with comorbid inhalant allergy using RAST for 15 index antigens. Patients were divided into short-term (0–6 months of allergy desensitization) and long-term (at least 12 months of allergy desensitization) groups, and their inner ear function was assessed with ECochG after intranasal challenge with a specific antigen. The results derived from this study are inconclusive, mainly because of the lack of a complete dataset. Still, the protocol developed by the authors was used in another paper published by Topuz et al. (46), who included five MD patients with inhalant allergy. Still, the sample was more homogeneous this time, as none of the patients were desensitized. The authors consistently observed increased SP/AP in all patients after nasal challenge with a patient-specific allergen. However, despite this increase, only one patient developed audio-vestibular symptoms. Topuz et al. (46) performed ECochG in 48 MD patients, separately for each ear (80 MD-affected and 16 MD-unaffected ears) immediately before and after the SPT. The SP/AP ratio indicative of endolymphatic hydrops was elevated in 23 MD-affected and three unaffected ears before the prick test and in 62 MD-affected and 13 unaffected ears after the prick test. A provocation test was performed in addition to the prick test, but the results were not different from those after the prick test. Although a control group of healthy subjects was lacking, these results support the role of antigenic stimulation in episodes of MD.

Howard et al. (47) evaluated the effect of anti-allergic therapy on otologic symptoms in 113 MD patients, where those with food allergies were treated by elimination of the allergenic factor, a rotating diet, or oral desensitization, and those with respiratory allergies were treated by desensitization to inhalant allergens. Compared to pre-treatment data, patients improved significantly 24 months after treatment in both allergic symptoms (improvement in runny nose, sore throat, eczema, pre-post comparison *p* < 0.001) and MD symptoms (decreased frequency of dizziness and tinnitus *p* < 0.005; decreased severity of dizziness, decreased tinnitus and unsteadiness per-post comparison, *p* < 0.001) and interference with daily activities (pre-post comparison *p* < 0.001). When compared to the non-desensitized control MD patients, the number of patients with significantly improved vertigo was greater in the treated MD group (*p* < 0.05). In comparison, the number with significantly worse vertigo was substantially lower (*p* < 0.001). There were fewer MD subjects with worse tinnitus in the treated group than in the untreated group (*p* < 0.001) and significantly fewer with allergy complaints (*p* < 0.01).

### 3.5 Presence of inner ear disorders in patients with allergies

Five studies (15, 56–59) focused on inner ear disorders in patients with allergies (Table 4). Three studies (56, 58, 59) showed that pure tone audiometry, otoacoustic emissions, and ABR were abnormal in patients with allergic rhinitis compared with healthy non-allergic controls, strongly suggesting inner ear pathology. One study (15) showed a high prevalence of various inner ear disorders in AR patients (see section 3.2).

**Table 4:** Studies on the audiological profile of patients with allergic diseases.

References	Study group	Control group	Inner ear examination	Main findings
Sahni et al. (58)	AR, *n* = 100	Exposed to a similar environment but were not suffering from AR, *n* = 100	Pure-tone audiometry, impedance audiometry, OAE	32 AR patients had SHL (4,000 and 8,000 Hz). 32 patients had abnormal OAE
Mahajan et al. (59)	AR, *n* = 100	Exposed to a similar environment but were not suffering from AR, *n* = 50	Pure-tone audiometry with extended high frequencies (0.250–12,000 Hz), OAE and ABR	SHL, abnormal ABR and abnormal DPOAE
Singh et al. (56)	AR, *n* = 30	Exposed to a similar environment but were not suffering from AR, *n* = 30	Pure-tone audiometry with extended high frequencies (0.250–16,000 Hz), OAE and ABR	SHL, abnormal TOAE and DPOAE, abnormal ABR
Lasisi and Abdullahi (15)	Nasal allergy, *n* = 144	None	Pure-tone audiometry (250–8,000 Hz)	66% of patients had otologic symptoms: 26.3% idiopathic tinnitus, 18% idiopathic SHL, 6% cochlear hydrops, and 6% autoimmune inner ear disease
Karabulut et al. (57)	AR patients with positive skin prick test, *n* = 58	AR patients with negative skin prick test, *n* = 31	Pure-tone audiometry at 250, 500, 1,000, 2,000, 4,000, and 8,000 Hz and immittance measures	AR patients had a lower pure-tone threshold in one frequency of 8,000 Hz

AR, allergic rhinitis; SHL, sensorineural hearing loss; OAE, otoacoustic emission; ABR, auditory brainstem response; DPOAE, distortion-product otoacoustic emission.

Finally, a study by Karabulut et al. (57) used pure-tone audiometry in a sample of AR patients and found a statistically significant difference between the hearing ability of 58 AR patients and 31 healthy controls at one frequency, 8,000 Hz, however, no differences were found for speech discrimination or other parameters tested.

## 4 Discussion

This work aimed to review the clinical evidence for an association between allergic diseases and inner ear disorders. Based on a search of two databases and inclusion and exclusion criteria, we identified 21 records suitable for our review. Most of the studies identified have focused on the prevalence and characterization of allergic symptoms in patients with inner ear disorders, namely MD, ISSHL, and ALHL. The remaining five publications examined inner ear disorders’ incidence or grade in patients with AR.

### 4.1 Epidemiological link between allergic diseases and inner ear disorders

The answer to our main question posed at the beginning of this work (“Is there a relationship between allergic diseases and inner ear disorders?”) is “yes.” All but two papers (49, 57) found supportive evidence of a possible association between allergy and inner ear disorders. The backing evidence included an increased prevalence of allergic disorders (airborne or food allergies) in persons with MD, ISSHL, and ALHL (48, 50–54) and an increased prevalence of inner ear disorders in AR patients (15, 56, 58, 59) when compared to the control groups or general population. In Derebery’s study (48), 41.6% of 734 unselected patients with MD reported having airborne allergies, 17.6% having suspected airborne allergies, and 40.3% having suspected food allergies. Wheat, milk, corn, egg, yeast, and soy were the most common food allergens identified in that study. Corroborating the results of Di Berardino and Cesarani (53) reported that 82.7% of MD patients (48/58) were positive for airborne or food allergens. Moreover, elevated levels of total and specific IgE were found in patients with MD, ISSHL and ALHL (31, 50, 54, 55) when compared to the controls, whereas the study by Ma et al. (31) found that total and specific IgE levels positively correlated with the SP/AP ratio and were predictors of ALHL recurrence and its transformation to MD.

In the first paper that did not provide supportive evidence for the link between allergies and inner ear disorders, the authors used serum samples from MD patients to investigate the levels of typical food allergen-specific IgG and IgA but did not explore the levels of total or specific IgE (49). Therefore, it cannot be concluded that the food allergy does not affect MD patients (66). The authors of the second paper used pure tone audiometry in the frequencies between 250 and 8,000 Hz, and found a difference in one frequency (8,000 Hz) between AR patients and healthy age-matched controls (57), suggesting that the AR patients have better hearing ability in that particular frequency. The authors speak of “hearing loss” in healthy subjects and the absence of hearing loss in AR patients. Nevertheless, these results should be interpreted with caution. The first reason for this is that the WHO definition of hearing loss is based on the average of the hearing thresholds at the four specific frequencies (500, 1,000, 2,000, and 4,000 Hz) measured in the “better” ear (67). However, Karabulut’s study compared the average for either 250 and 500 Hz, 500, 1,000, and 2,000 Hz, or 4,000 and 8,000 Hz; therefore, the WHO standards cannot be applied. The second reason is that the authors defined hearing loss differently from the WHO (67), namely as greater than 15 dB, whereas the WHO defines it as greater than 20 dB in the better ear and greater than 35 dB in the worse ear.

### 4.2 Inner ear disorders associated with allergies

In search of an answer to our second question (in which inner ear disorders have the allergic disorders been observed and vice versa), we identified three inner ear disorders that have been studied in relation to allergy: MD, ISSHL, and ALHL. All three are rare, heterogenous, and lacking animal models, which are the reasons for the many blank spots on the map of their pathogenesis. Because of the heterogeneity of MD (e.g., differences in the number of episodes per week, year, or lifetime; variable duration; laterality), many attempts have been made to classify it. One of the recent classifications divided unilateral MD into five subtypes (68), but the presence or absence of allergies, although noted by the authors as worthy of further research, was not used in this classification. Allergy (presence of hay fever) was considered an essential factor associated with MD [odds ratio (OR) 3.1] in the cluster analysis, which identified two clusters of MD and has associated hay fever with cluster 2, along with low-frequency tinnitus, stress-induced vertigo, bilateral tinnitus, depression, autoimmune disease, drug intolerance, and migraine (69). In addition, a recent review on allergy and MD, published as we were preparing our work for submission, explored this issue in depth and confirmed the importance of allergy in the diagnosis and etiology of MD (70). Furthermore, a recent meta-analysis identified allergies (defined as asthma, allergic asthma, and AR) as important risk factors for MD (OR = 2.27) (71).

Much less is known about the association of allergies with both types of sudden hearing loss: ISSNHL and ALHL. Apart from the evidence collected, we found a recent paper showing that the risk of developing ISSNHL is higher in patients with asthma than in controls (72). Although this was a longitudinal follow-up study using a retrospective cohort and health insurance records, and no information was provided on the type of asthma, based on other studies (73), it can be assumed that about 50% of the asthma cases were due to type I hypersensitivity disorders. No new research could be identified at the beginning of 2024, reflecting the rarity of both diseases and either lack of interest or resources in the research community. However, it would be very interesting to confirm or deny such an association since some of the ALHL patients progress to developing full-blown MD (30). The open question is whether allergy increases the odds ratio for such transformation and, if so, whether timely allergy treatment could prevent progression to MD.

### 4.3 Effects of allergic modulation on the inner ear function

In response to our third question (“How does the hearing or vestibular system respond to treatment of allergic diseases in comorbid patients?”), we found evidence that systemic allergic provocation induces cochlear fluid changes in MD patients consistent with endolymphatic hydrops (41, 42, 44, 45, 74). Interestingly, some but not all MD patients responded to the allergic provocation test with vertigo and a sense of fullness in the cochlea (46). Moreover, the most clinically interesting phenomenon, namely successful desensitization in MD patients, was associated with a decrease in the number of MD episodes and an improvement in the patient’s quality of life (43, 47).

### 4.4 Effects of allergy on the middle ear function

This review focused strictly on sensorineural hearing loss, defined as a pathology that causes hearing loss and involves either the cochlea, vestibulocochlear nerve, auditory brainstem, or central auditory structures. However, the hearing problems commonly reported in people with allergies are caused by conductive hearing loss due to middle ear disorders, which were not the focus of this review. The two types of hearing loss (conductive and sensorineural) can be distinguished by otoscopy, fork tests such as the Rinne test (75), the Weber test (76), and bone- and air-conduction pure tone audiometry (77, 78). Conductive hearing loss associated with allergy can often be caused by Eustachian tube obstruction due to allergic edema of the mucosa (79). Another cause of allergic conductive hearing loss may be otitis media facilitated by allergic processes (80). Finally, chronic suppurative otitis media (CSOM), a persistent inflammatory disease often associated with allergy and the most common cause of acquired hearing loss in developing countries (81) can lead to both, conductive and sensorineural hearing loss (82).

### 4.5 Possible mechanisms involved in allergy-mediated inner ear disorders

**Figure 2:**
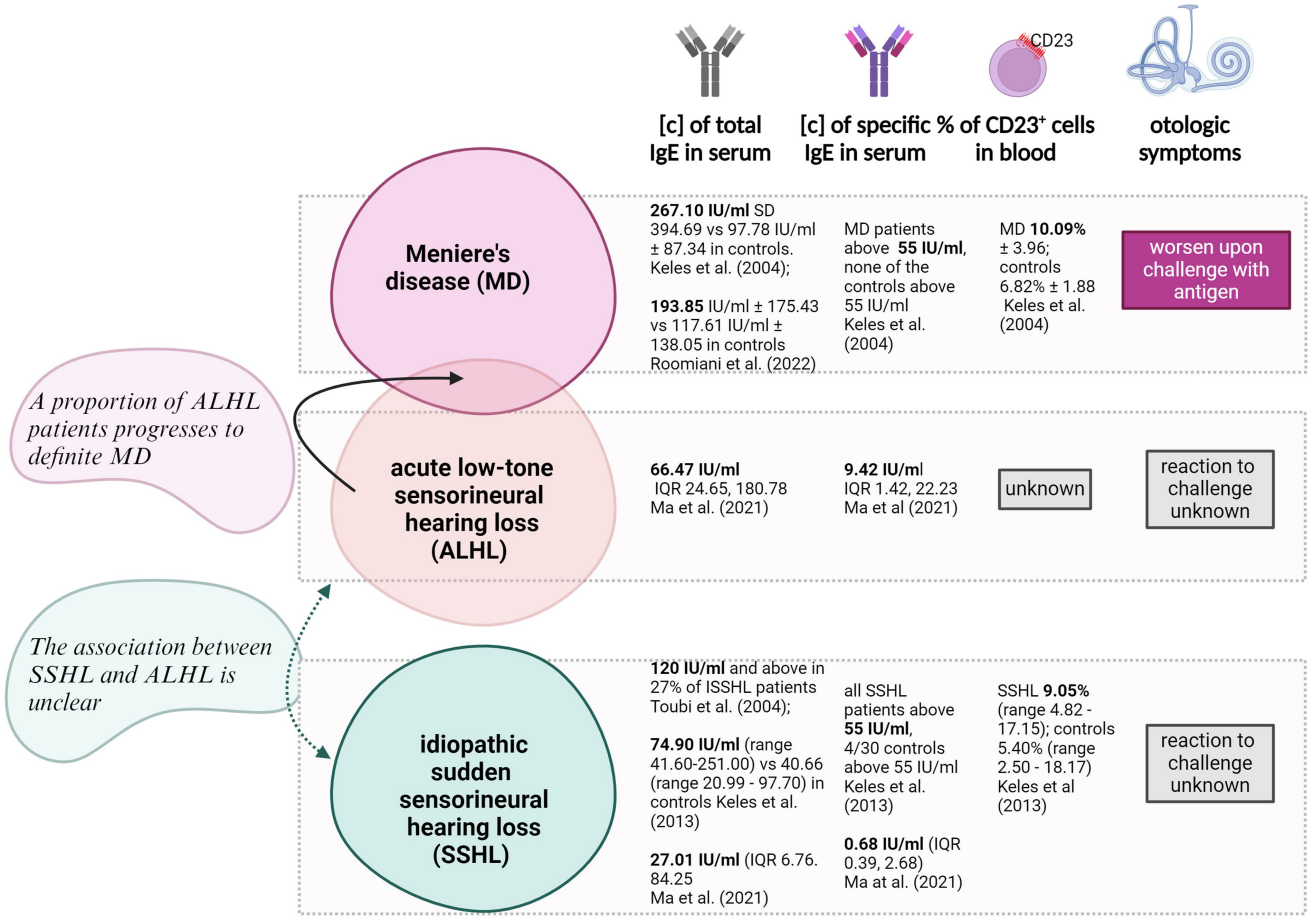
Schematic presentation of key data mined in this paper the presence of type I hypersensitivity-associated symptoms in patients affected by one of three auditory disorders: MD (Meniere’s disease), ALHL (acute low-tone hearing loss) and idiopathic SSHL (sudden sensorineural hearing loss). Four parameters were included: the concentration of total IgE in serum measured by ELISA, the concentration of specific IgE in serum measured by ELISA, the percentage of CD23+ cells in the circulation and the changes in otologic symptoms in reaction to antigenic challenge. IU, international units; IQR, interquartile ranges; [c], concentration. Created with BioRender.com.

The clinical observations reviewed here provide only some clues as to the mechanisms by which the allergic disease may induce ISSNHL, ALHL, and MD (Figure 2), including increased serum total (ISSNHL, ALHL, and MD) and specific (ALHL and MD) IgE concentrations; increased numbers of circulating CD23+ cells (ISSNHL and MD); the ability of antigen challenge to provoke disease symptoms (MD); and finally, some efficacy of desensitization in reducing disease symptoms (MD).

Complementary information was provided by *in vitro* studies using dissected vestibular apparatus, which showed significantly higher levels of IgE and CD23 at protein and mRNA levels in MD patients than in vestibular schwannoma patients (83). The same study has shown that CD23 expressed on the polarized murine hair cell line HEI-OC1 effectively transports IgE across the cell (83) and that IL-4 accelerates this transport by increasing CD23 expression. The phenomenon of IgE transcytosis by CD23 has been documented in other types of human epithelial tissue, such as respiratory (84) or intestinal (85) epithelia, and blocking IgE transcytosis inhibits the onset and progression of allergic airway inflammation in mice (86). In addition, the levels of proinflammatory/proallergic cytokines (IL-4, IL-5, IL-10, and IL-13) were higher in the macula, ampulla, and endolymphatic sac dissected from patients with MD than in the specimens from patients with vestibular schwannoma (83). A recent study performed protein analysis at the single cell level using flow cytometry-based clustering of blood cells from MD patients based on granulocyte/lymphocyte ratio (high and low). They found that stimulation of peripheral blood cells isolated from MD patients with a common mold or bacterial antigen produced significantly more IL-4 and IL-6 in patients with a high granulocyte/lymphocyte ratio compared to the MD group with a low ratio or healthy controls (87). The preferential production of IL-4 and IL-6 indicates the creation of Th2-friendly environment during immune responses and in particular that involved in isotype switch and IgE production by B/plasma cells (88). Finally, studies using animal models demonstrated that a local antigen sensitization can induce symptoms such as nystagmus, endolymphatic hydrops or hearing loss (89–92).

### 4.6 Clinical implications

Our literature review suggests the need for specialized allergy testing in patients with MD, ISSHL, and ALHL. Identification of patients with allergic features can be used as a basis for specific anti-allergy therapy (primary outcome) with secondary outcome being inner ear symptoms. This type of therapy was used by Derebery in form of allergen desensitization with the secondary outcome MD symptoms (43). Desensitization is only one of the currently available treatments for allergies, among which biologic therapies have begun to dominate in recent years. One of the biologic therapies (dupixent/dupilumab) inhibits IL-4 and IL-13 signaling by specifically binding to the alpha subunit of the IL-4 receptor. Elevated levels of both interleukins have been found in stimulated peripheral blood cells of patients with MD and an increased granulocyte/lymphocyte ratio. Therefore, it can be speculated that in this type of patient, the use of dupixent/dupilumab may effectively reduce the signaling of both cytokines, which is potentially important in the progression of MD. Another biological drug, omalizumab, inhibits the binding of IgE to the high- and low-affinity receptors expressed on eosinophils, mast cells, and basophils and thus blocks IgE-induced signaling. Interestingly, in all inner ear diseases included in the current review study, serum IgE levels (non-specific or specific) were higher than in the control groups. Admittedly, this does not necessarily mean that IgE is crucial in all patients with MD, ISSHL or ALHL, but it may be in some.

Supporting this line of thought is a published clinical case of a patient with NSAID-exacerbated respiratory disease in whom sensorineural hearing loss and tinnitus unexpectedly resolved after therapy with dupilumab directed at respiratory symptoms (asthma and rhinosinusitis with polyps) (93). Another case report found a successful use of omalizumab for otologic symptoms in a patient with MD and cutaneous mastocytosis (94). Given the extremely small number of papers on the subject, it remains to be determined in what type of patients this type of therapy can be used.

### 4.7 Study limitations

One of the significant pitfalls of our review is that we did not systematically assess levels of evidence or extract meta-analytic data. This is due to the wide variation in studies, reflecting different standards of study design and reporting in the 1990s and the first decade of this century when the majority of the included manuscripts were published, as opposed to contemporary design. Another drawback was the small number of studies that met our inclusion criteria, exposing that MD, ISSHL, and ALHL are all rare and non-life-threatening diseases.

## 5 Conclusion

This systematic review summarizes the clinical evidence linking respiratory and food allergy to inner ear diseases such as MD, ISSHL and ALHL. Our findings suggest the possible existence of immune factors in the pathogenesis of inner ear conditions, and that allergy may be a likely source of inflammation in patients with inner ear disease. Further clinical research on the role of the immune system in the etiopathogenesis of inner ear diseases is needed to establish a definitive link between the two diseases, to improve the classification of inner ear diseases and to apply new treatments.

## Author contributions

BZ: Data curation, Formal analysis, Investigation, Visualization, Writing – original draft, Writing – review & editing. ED: Formal analysis, Investigation, Writing – review & editing. LK: Formal analysis, Investigation, Writing – review & editing. HO: Resources, Writing – review & editing. JS: Writing – review & editing, Project administration, Supervision. SMR: Writing – review & editing, Project administration, Supervision. FS: Writing – review & editing, Project administration, Supervision. AJS: Conceptualization, Formal analysis, Methodology, Visualization, Project administration, Resources, Supervision, Writing – review & editing.
